# Tubercidin inhibits PRRSV replication via RIG-I/NF-κB pathways and interrupting viral nsp2 synthesis

**DOI:** 10.1128/spectrum.03479-23

**Published:** 2024-02-01

**Authors:** Yuqian Xu, Zhenbang Zhu, Meng Zhang, Lulu Chen, Kegong Tian, Xiangdong Li

**Affiliations:** 1 Jiangsu Co-innovation Center for Prevention and Control of Important Animal Infectious Diseases and Zoonoses, College of Veterinary Medicine, Yangzhou University, Yangzhou, China; 2 National Research Center for Veterinary Medicine, Luoyang, China; 3 College of Veterinary Medicine, Henan Agricultural University, Zhengzhou, China; 4 Joint International Research Laboratory of Agriculture and Agri-Product Safety, the Ministry of Education of China, Yangzhou University, Yangzhou, China; Institute of Microbiology Chinese Academy of Sciences, Beijing, China

**Keywords:** PRRSV, tubercidin, antiviral activity, nsp2, cytokines

## Abstract

Porcine reproductive and respiratory syndrome virus (PRRSV) is an RNA virus with constantly emerging recombinant and mutant strains. Because of the high genetic diversity of PRRSV, current vaccines only provide partial protection against the infection of heterologous strains, which makes it a challenge for PRRSV prevention and control. Tubercidin is a naturally extracted compound with potential antiviral properties. However, whether tubercidin has anti-PRRSV ability is unknown. Our study found that tubercidin showed effective antiviral effects on PRRSV replication. In terms of mechanism, tubercidin suppressed PRRSV at the entry, replication, and release steps of the viral life cycle. Additionally, we demonstrated that tubercidin treatment promoted the activation of retinoic acid-inducible gene I and nuclear factor kappa-light-chain-enhancer of activated B cell signaling pathway, thus increasing the type I interferon and inflammatory cytokine expression. Furthermore, tubercidin restrained the viral non-structural protein 2 expression and viral dsRNA synthesis and ultimately inhibited PRRSV replication. Hence, our data showed that tubercidin is promising and has potential antiviral ability against PRRSV replication *in vitro*.

**IMPORTANCE:**

Porcine reproductive and respiratory syndrome (PRRS) is one of the most important swine diseases, which causes huge economic loss worldwide. However, there is no effective therapeutic method for PRRS prevention and control. Here, we found that tubercidin, a naturally extracted adenosine analog, exhibited strong anti-porcine reproductive and respiratory syndrome virus (PRRSV) activity. Mechanically, tubercidin inhibited viral binding, replication, and release. Tubercidin suppressed PRRSV non-structural protein 2 expression, which is important for the formation of replication and transcription complex, leading to the block of viral RNA synthesis and PRRSV replication. Moreover, tubercidin could activate retinoic acid-inducible gene I/nuclear factor kappa-light-chain-enhancer of activated B cell innate immune signaling pathway and increased the expression of interferons and proinflammatory cytokines, which was the other way to inhibit PRRSV replication. Our work evaluated the potential value of tubercidin as an antiviral agent on PRRSV replication and provided a new way to prevent PRRSV replication *in vitro*.

## INTRODUCTION

Porcine reproductive and respiratory syndrome (PRRS) has been one of the most important swine diseases since it emerged in North America two decades ago, which caused an enormous economic loss annually in the swine industry worldwide (1, 2). PRRS is characterized by reproductive disorder in sows, respiratory disease in swine of all ages, and mortality of newborn piglets (3). Porcine reproductive and respiratory syndrome virus (PRRSV), belonging to the family *Arteriviridae*, order *Nidovirales* (4), is the etiologic agent of PRRS. It is an enveloped positive single-stranded RNA virus with the genome RNA containing 11 known open reading frames (ORFs) (5). The replicase genes, ORF1a and ORF1b, encode pp1a and pp1ab polyproteins, which are cleaved into at least 14 non-structural proteins (nsps) that assemble to form a membrane-bound replication and transcription complex (RTC), initiating the synthesis of minus-stranded RNA (6, 7). Meanwhile, the RNA polymerase infidelity and the base modification contribute to the rapid recombination and evolution of PRRSV (8). Since PRRS has long been one of the swine pandemics across the world, numerous efforts have been made to control its infection. However, the current commercial PRRSV vaccine cannot provide complete protection for different strains, making the subject of controlling PRRSV remain a challenge (9, 10). Thus, there is an urge to develop efficacious vaccines or therapies against PRRSV.

Tubercidin is an adenosine analog obtained from *Streptomyces tubercidicus* and the cyanophyte *Tolypothrix byssoidea* (11, 12). It was also reported to be isolated from the marine sponge *Callyspongia biﬂabellata* (13). Tubercidin has diverse biological properties, including antiviral, antitumor, antiparasitic, and antibacterial abilities. In recent years, tubercidin has been demonstrated to be able to attenuate SARS-CoV-2 and influenza B virus (IBV) proliferation (14, 15) and showed anti-small-cell lung cancer ability (16). In addition, tubercidin had strong antiparasitic activity against *Trypanosoma brucei* (17, 18), *Trypanosoma gambiense* (19), *Trypanosoma congolense* (18), *Schistosoma mansoni*, *Schistosoma japonicum* (20), and *Leishmania* spp. (21, 22). It also showed antibiotic capacity against *Candida albicans, Mycobacterium tuberculosis, and Streptococcus faecalis* (23). However, whether tubercidin has antiviral effects on PRRSV remains unknown. In this study, we evaluated the antiviral activity of tubercidin on PRRSV replication *in vitro* and demonstrated the underlying mechanism that tubercidin blocked viral non-structural protein 2 (nsp2) synthesis and facilitated the activation of retinoic acid-inducible gene I (RIG-I)/nuclear factor kappa-light-chain-enhancer of activated B cell (NF-κB) signaling pathway. Our work sheds light on a potential therapy for PRRSV control.

## MATERIALS AND METHODS

### Cells, viruses, and tubercidin preparation

Marc-145 cells (a PRRSV-permittable cell line, which originated from African green monkey kidney cell line MA-104) and HEK293T cells were cultured in Dulbecco’s modiﬁed Eagle medium (DMEM) (Gibco, USA) with 10% fetal bovine serum (FBS) (Gibco, USA) at 37°C in 5% CO_2_. Porcine alveolar macrophages (PAMs), the primary target cells of PRRSV, were harvested from swine alveolar lavage fluid with phosphate-buffered saline (PBS) and maintained in RPMI 1640 medium (Gibco, USA) containing 10% FBS at 37°C in 5% CO_2_. There were four strains used in the study, namely, CHR6, XJ17-5, Li11, and SD16. CHR6 and Li11 were preserved in our laboratory. XJ17-5 and SD16 were generously provided by Nanhua Chen from Yangzhou University. PRRSV titers were defined by 50% tissue culture infective dose (TCID_50_). Tubercidin was purchased from the manufacturer (MedChemExpress, USA) and diluted in dimethyl sulfoxide at a concentration of 1 µM and stored at −20°C.

### Cytotoxicity assay

The enhanced Cell Counting Kit-8 (CCK-8) (Beyotime, Shanghai, China) was used to determine the viability of Marc-145 cells and PAMs after tubercidin treatment. Cells were seeded in 96-well plates treated with different concentrations of tubercidin for 36 or 48 h and incubated with 10% CCK-8 solution in the dark at 37°C for another 2 h. The absorbance was detected at 450 nm indicating the cytotoxicity of tubercidin treatment.

### Quantitative real-time reverse transcription polymerase chain reaction

Total intercellular RNA was obtained from Marc-145 cells and PAMs using TRIzol reagent (TIANGEN, Beijing, China) for quantitative real-time reverse transcription polymerase chain reaction (RT-qPCR). cDNA was inverse-transcribed from 1 µg of RNA using HiScript IIIRT SuperMix (Vazyme, Nanjing, China) according to the instructions of the manufacturer. The cDNA products were amplified with the following primers and a ChamQ Universal SYBR qPCR Master Mix (Vazyme, Nanjing, China) using ABI QuantStudio3 (Applied Biosystems, Waltham, MA, USA). The primers used in this study are listed in Table 1. The reaction program was conducted as follows: 95°C for 1 min, followed by 40 cycles at 95°C for 5 s and 60°C for 1 min. The relative expression of the genes above was calculated using the 2^−ΔΔCt^ method and normalized to GAPDH or HTPR1.

**TABLE 1:** Primers used for RT-qPCR

Primer^*a*^	Sequence (5′−3′)^*b*^
PRRSV N-F	AAAACCAGTCCAGAGGCAAG
PRRSV N-R	CGGATCAGACGCACAGTATG
pIFN-β-F	AGCACTGGCTGGAATGAAACCG
pIFN-β-R	CTCCAGGTCATCCATCTGCCCA
pISG56-F	GCGCTGGGTATGCGATCTC
pISG56-R	CAGCCTGCCTTAGGGGAAG
pIL-1β-F	CCCAAAAGTTACCCGAAGAGG
pIL-1β-R	TCTGCTTGAGAGGTGCTGATG
pIL-6-F	CTGCTTCTGGTGATGGCTACTG
pIL-6-R	GGCATCACCTTTGGCATCTT
pIL-8-F	AGTTTTCCTGCTTTCTGCAGCT
pIL-8-R	TGGCATCGAAGTTCTGCACT
pTNF-α-F	ACTCGGAACCTCATGGACAG
pTNF-α-R	AGGGGTGAGTCAGTGTGACC
pHPRT1-F	TGGAAAGAATGTCTTGATTGTTGAAG
pHPRT1-R	ATCTTTGGATTATGCTGCTTGACC
mGAPDH-F	TGACAACAGCCTCAAGATCG
mGAPDH-R	GTCTTCTGGGTGGCAGTGAT

^*a*^ F, forward primer; R, reverse primer. The letter “p” represents a pig gene; the letter “m” represents a green monkey gene.

^*b*^ Pig gene sequences, green monkey gene sequences, and PRRSV gene sequences were downloaded from GenBank.

### Western blot

Cells, after being washed three times with PBS, were lysed with lysis buffer (Beyotime, Shanghai, China) for 20 min on ice and collected for the bicinchoninic acid method (Beyotime, Shanghai, China) to quantify the lysates. Protein lysate samples were subjected to SDS-PAGE and transferred to a polyvinylidene difluoride membrane (Merck Millipore, Billerica, MA, USA). After being blocked by 5% fat-free milk (Sangon Biotech, Shanghai, China) in TBST (20 mM Tris-HCl pH 8.0, 150 mM NaCl, 0.05% Tween 20) for 2 h at room temperature, membranes were incubated with indicated protein antibodies diluted in a ratio of 1:1,000 at 4°C overnight. The antibodies used in the study include anti-PRRSV N (MEDIAN, Republic of Korea) antibody, anti-GAPDH, RIG-I, MDA5, mitochondrial antiviral-signaling protein (MAVS), p65, phosphorylated p65, IRF3, phosphorylated IRF3, phosphorylated IκBα (Cell Signaling Technology, Danvers, MA, USA) antibodies, and anti-nsp2 antibody was provided by Prof. Yanhua Li from Yangzhou University. The membranes were incubated with corresponding secondary antibodies diluted in a ratio of 1:8,000 for 1 h and then washed with TBST three times. The protein signals were visualized using an enhanced chemiluminescence reagent (NCM Biotech, Suzhou, China) on a Tanon 5200 system (Tanon, Shanghai, China).

### Immunofluorescence assay

Marc-145 cells were cultured in 12-well plates and treated with tubercidin and PRRSV at the indicated time points. Then, cells were fixed with 4% paraformaldehyde (Biosharp, China) for 10 min and permeabilized with 0.5% Triton X-100 (Solarbio, China) for 15 min at room temperature. After being washed by PBS three times, cell samples were blocked by 3% bovine serum albumin for 30 min at 37°C. Then, cells were incubated with primary antibodies at 4°C overnight. Next, cells were cultured with indicated secondary antibodies for 1 h avoiding exposure to light and incubated with 4′,6-diamidino-2-phenylindole dihydrochloride (DAPI) (Beyotime, Shanghai, China) for another 5 min. The fluorescence of the target proteins was observed using a confocal laser scanning microscope (TCS SP8 STED; LEICA, Wetzlar, Germany) or an inverted fluorescence microscope (UHGLGPS; Olympus, Tokyo, Japan). The primary antibodies used in immunofluorescence assay (IFA) included an anti-PRRSV N antibody, an anti-NF-κB p65 antibody, an anti-dsRNA antibody (Scicons, Szirák, Hungary), and an anti-nsp2 antibody.

### Viral binding, entry, replication, and release assays

For the binding assay, Marc-145 cells were seeded in 6-well plates in DMEM with 1% FBS at 4°C for 2 h and then infected with PRRSV [multiplicity of infection (MOI) = 5] with indicated concentrations of tubercidin at 4°C for 4 h. Cells were collected to extract the total RNA for further detection.

For the entry assay, Marc-145 cells were cultured at 4°C for 2 h and infected with PRRSV (MOI = 5) at 4°C for 4 h. After being washed with cold PBS, cells were treated with mentioned concentrations of tubercidin at 37°C for 4 h. Cells were washed with a solution of high-alkaline and high-salt solution [1 M NaCl and 50 mM sodium bicarbonate (pH 9.5)] and harvested to examine the PRRSV mRNA expression using RT-qPCR.

For the replication assay, Marc-145 cells were infected with PRRSV (MOI = 1) in the absence or presence of tubercidin for 12, 24, and 36 h. RT-qPCR was used to determine the mRNA expression of PRRSV ORF7.

For the release assay, Marc-145 cells were infected with PRRSV at an MOI of 1 for 12 h, and the supernatants of cells were removed. Then, 1% DMEM and indicated tubercidin were added into the cells at 37°C for another 12 h, and the supernatants were collected for TCID_50_ assay.

### Detection of interferon and inflammatory cytokine genes

To investigate the relative mRNA expression of IFN-β, interleukin-1β (IL-1β), IL-6, IL-8, tumor necrosis factor-α (TNF-α), and ISG56, PAMs were infected with PRRSV (MOI = 1) in the absence or presence of tubercidin, and then, cells were collected to detect their relative expression using RT-qPCR. The data were normalized to HPRT1.

### Expression vector construction and transfection

PRRSV nsp2 gene was ampliﬁed from the PRRSV strain and cloned into vector mCherry-N (632523, TaKaRa, Japan) with an N-terminal mCherry tag. HEK293T cells were seeded in 6-well plates and transfected with 3,000 ng of nsp2-mCherry plasmids using Lipofectamine 2000 (Invitrogen, Carlsbad, CA, USA) for 24 h in the absence or presence of tubercidin. Finally, cells were collected for western blot analysis.

### Statistical analysis

GraphPad Prism 9.0 was utilized for the data analysis with one- or two-way analysis of variance. Data are presented as mean ± SEM. The different levels of significance are presented as follows: (*) *P* < 0.05, (**) *P* < 0.01, (***) *P* < 0.001.

## RESULTS

### Tubercidin inhibits PRRSV replication in Marc-145 cells and PAMs

**Fig 1:**
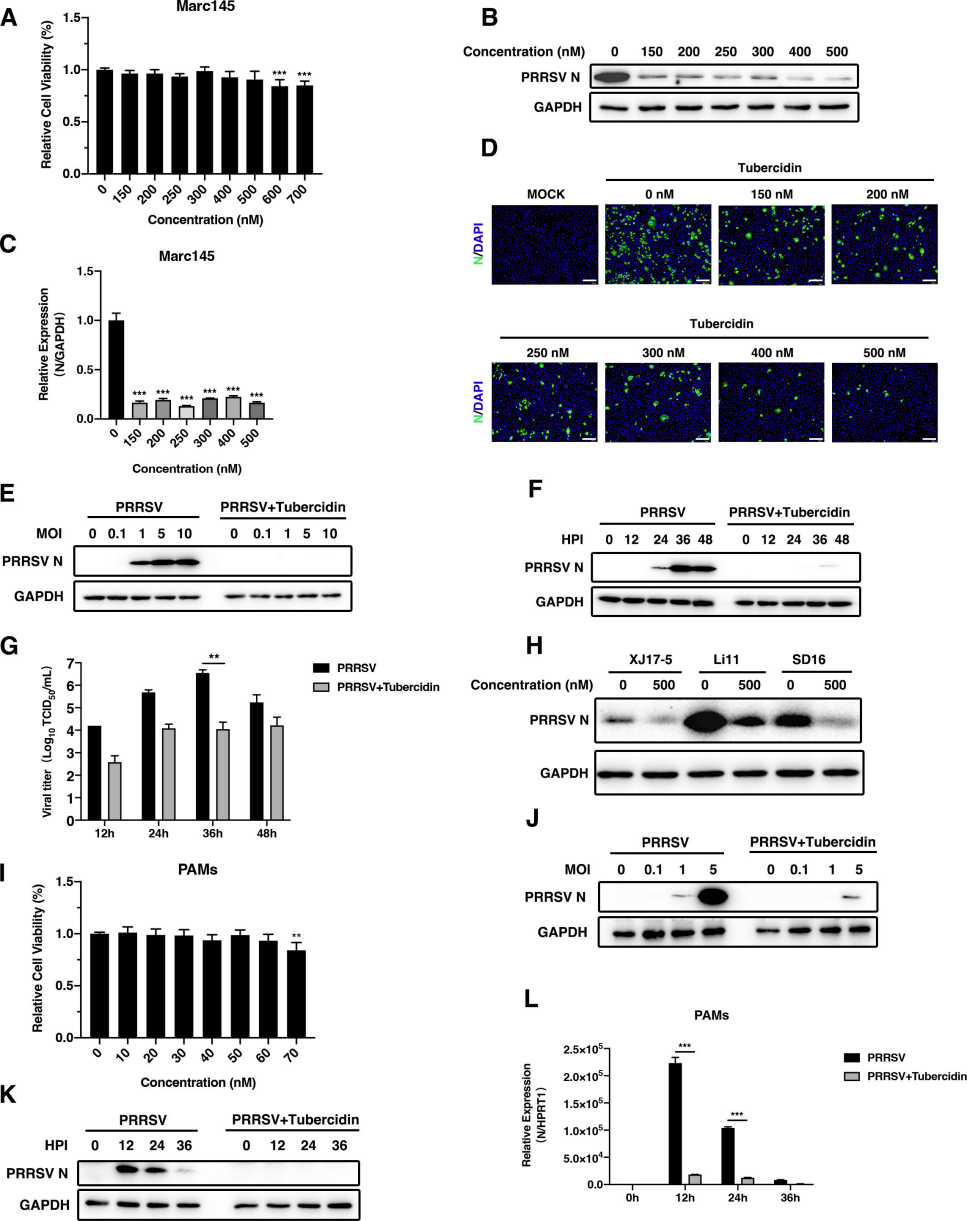
Tubercidin inhibits PRRSV replication in Marc-145 cells and PAMs. (A) The cytotoxicity of tubercidin in Marc-145 cells was measured by CCK-8 assay. Marc-145 cells were treated with the mentioned concentrations of tubercidin for 48 h, and a cell viability assay was performed. (**B through D**) Marc-145 cells were infected with CHR6 PRRSV strain (MOI = 1) in the presence of tubercidin (0, 150, 200, 250, 300, 400, 500 nM) for 24 h. The expression of PRRSV N was analyzed by western blot (B), RT-qPCR (C), and IFA (D). Scale bar, 200 µm. (E) Marc-145 cells were infected with CHR6 strain at different MOIs in the presence (500 nM) or absence of tubercidin. N protein was determined by western blot. (**F and G**) Marc-145 cells were infected with CHR6 strain (MOI = 1) in the absence or presence of tubercidin (500 nM) and harvested at indicated time points. The expression of the N protein was measured by western blot (F), and the cell supernatants were collected to detect viral titers using TCID_50_ assay (G). (H) Marc-145 cells were infected with XJ17-5 strain, Li11 strain, and SD16 strain (MOI = 1) in the presence (500 nM) or absence of tubercidin for 24 h. PRRSV N protein was determined by western blot. (I) The cytotoxicity of tubercidin in PAMs was measured by CCK-8 assay. (J) PAMs were infected with CHR6 strain at different MOIs mock treated or treated with tubercidin (50 nM) and then harvested to detect the expression of N protein using western blot. (**K and L**) PAMs were infected with CHR6 strain (MOI = 1) and harvested at different time points. Western blot (K) and RT-qPCR (L) were used to analyze the PRRSV N expression. Significant differences are denoted by (*) *P* < 0.05, (**) *P* < 0.01, (***) *P* < 0.001.

To determine the appropriate concentrations of tubercidin for the following experiments, we ﬁrst tested the cytotoxicity of tubercidin in Marc-145 cells using CCK-8 assay. As shown in Fig. 1A, the cell viability of Marc-145 did not show a significant difference within the concentrations ranging from 150 to 500 nM. Then, we examined the antiviral ability of tubercidin against CHR6 PRRSV strain in Marc-145 cells using western blot, RT-qPCR, and IFA (Fig. 1B through D) at 24 hpi. As shown in Fig. 1B and C, tubercidin at different concentrations downregulated the mRNA and protein expression levels of PRRSV N compared to the cells without tubercidin treatment. The fluorescence of PRRSV N (green) was reduced at concentrations of tubercidin ranging from 0 to 500 nM (Fig. 1D). Moreover, we found that tubercidin considerably reduced the expression of N protein in dose- and time-dependent manners (Fig. 1E and F). Besides, the viral titer with tubercidin treatment was noticeably lower than that of CHR6 PRRSV infected alone at 36 h (Fig. 1G). Next, we tested the N protein expression of XJ17-5 strain, Li11 strain, and SD16 strain in the presence or absence of tubercidin to analyze whether the drug can exert an antiviral effect against different PRRSV strains. As expected, tubercidin reduced the N protein expression of distinct strains at different degrees, indicating that tubercidin inhibited different PRRSV strain replications (Fig. 1H). Additionally, tubercidin, at a maximum concentration of 60 nM, did not impair PAM viability compared with the control (Fig. 1I). Furthermore, a similar dose- and time-dependent decrease in PRRSV N expression upon tubercidin treatment could be observed in the PAMs (Fig. 1J and K). Meanwhile, the relative expression level of ORF7 was reduced sharply at 12 and 24 hpi compared with the untreated cells (Fig. 1L). These results demonstrated that tubercidin exhibited an inhibitory effect on PRRSV replication *in vitro*.

### Tubercidin exerts an inhibitory effect on PRRSV replication at three different administration routes

**Fig 2:**
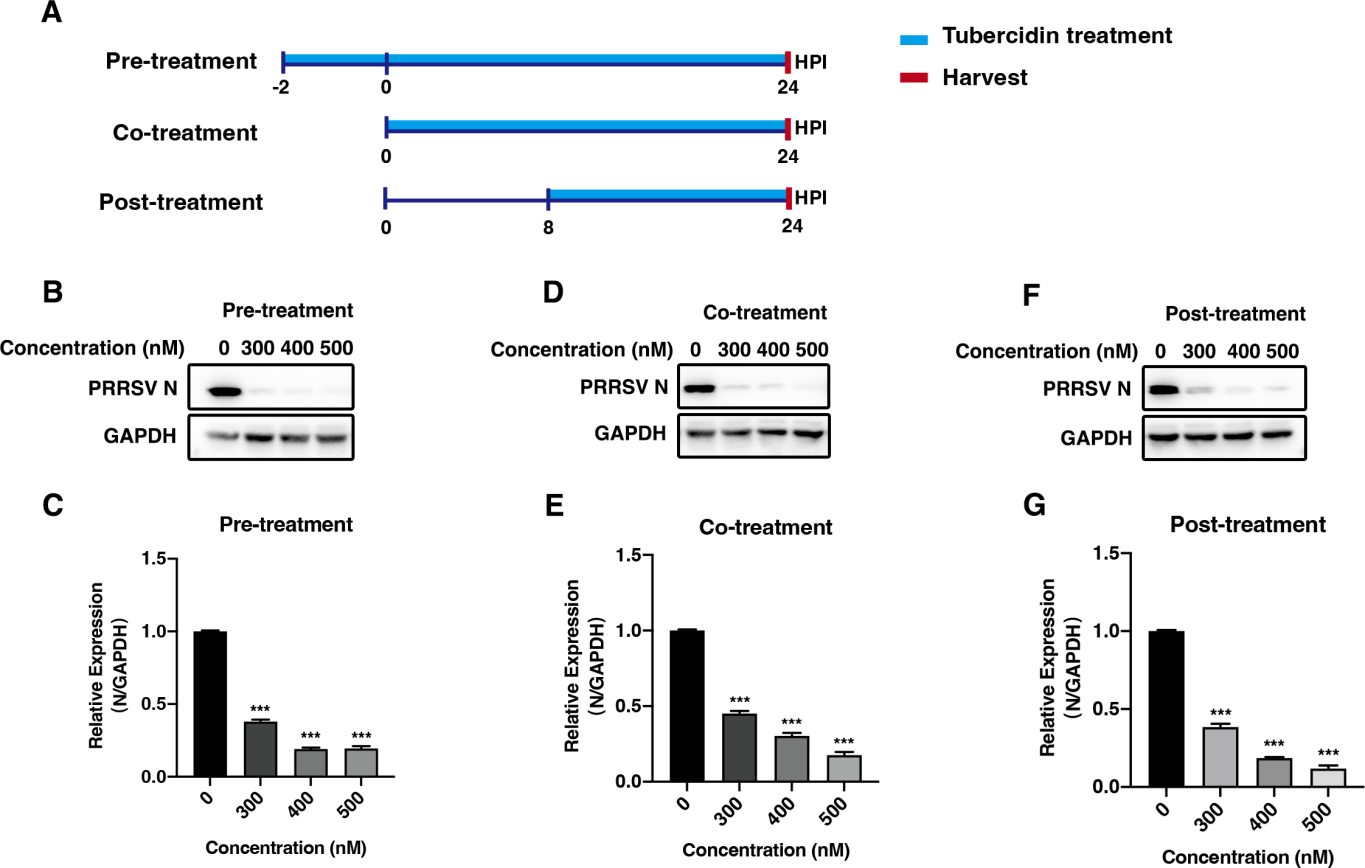
Tubercidin exerts an inhibitory effect on PRRSV replication at three different administration routes. (A) The diagram of three different administration treatment modes. (**B and C**) Marc-145 cells were pre-treated with different concentrations of tubercidin (0, 300, 400, 500 nM) for 2 h and then infected with CHR6 strain (MOI = 1) for another 24 h. The expression of PRRSV N was detected by western blot (B) and RT-qPCR (C). (**D and E**) Marc-145 cells were infected with CHR6 strain (MOI = 1) and incubated with tubercidin (0, 300, 400, 500 nM) for 24 h and harvested using western blot (D) and RT-qPCR (E) to determine PRRSV N and viral ORF7 expression. (**F and G**) Marc-145 cells were infected with CHR6 strain at an MOI of 1 for 8 h, then treated without or with tubercidin (300, 400, 500 nM) for another 24 h, and collected to analyze N protein (F) and ORF7 mRNA expression (G). Significant differences are denoted by (*) *P* < 0.05, (**) *P* < 0.01, (***) *P* < 0.001.

Since tubercidin could block PRRSV replication, we further examined whether it could inhibit PRRSV replication at different administration routes. The diagram in Fig. 2A displays the design of the following experiments. Marc-145 cells were infected with PRRSV, accompanied by pre-, co-, or post-treatment with tubercidin at concentrations of 300, 400, and 500 nM. As shown in Fig. 2B and C, tubercidin significantly reduced the N protein expression and ORF7 relative mRNA expression in the way that Marc-145 cells were pre-treated with tubercidin for 2 h before virus infection. Moreover, co- and post-treatment exhibited similar inhibitory effects, manifesting a decrease in viral N protein and ORF7 mRNA expression (Fig. 2D through G). These data indicated that tubercidin showed obvious inhibition on PRRSV proliferation no matter what kind of treatment.

### Tubercidin blocks the entry, replication, and release of PRRSV but does not influence PRRSV attachment

**Fig 3:**
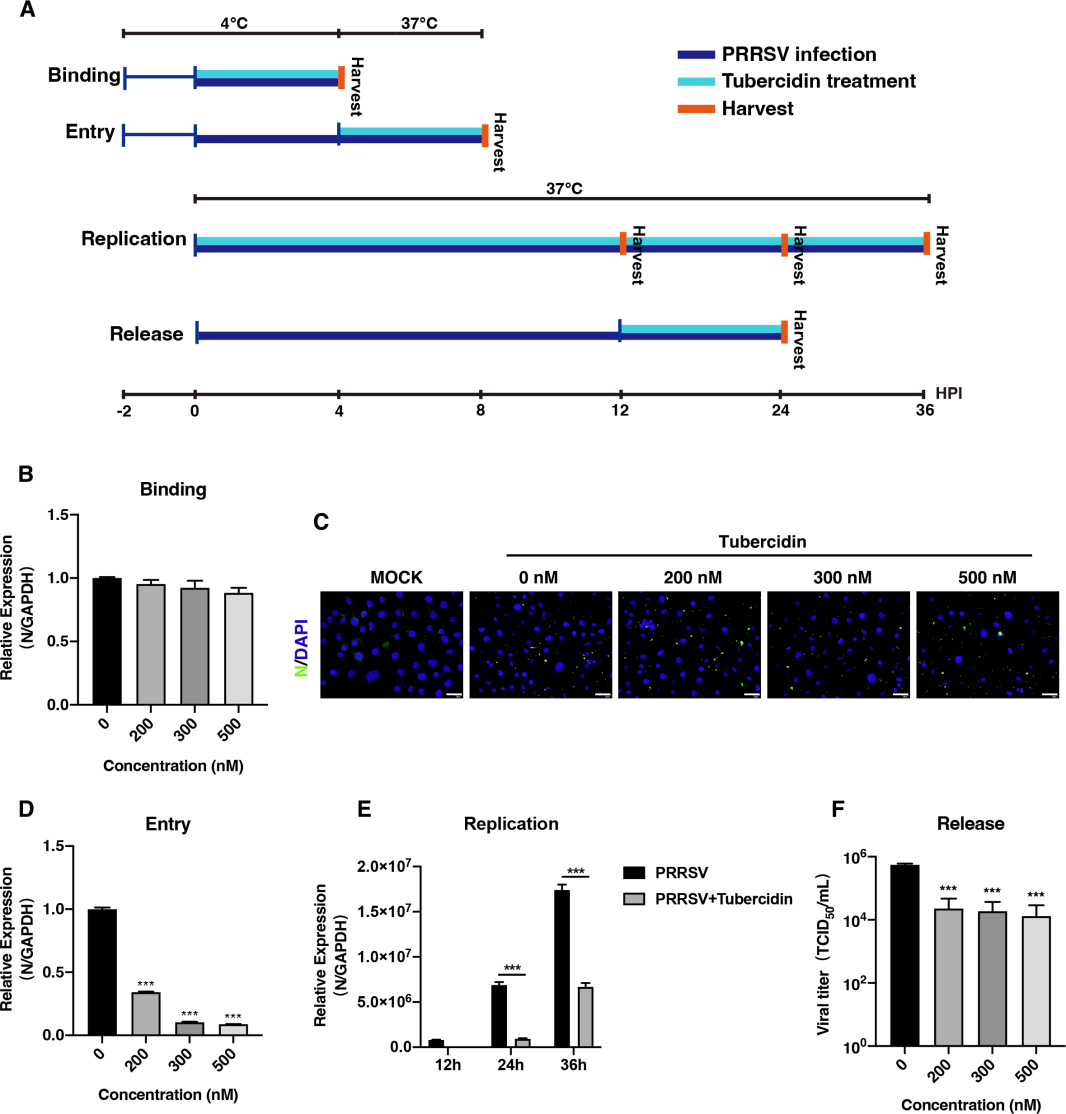
Tubercidin blocks the entry, replication, and release of PRRSV but does not influence PRRSV attachment. (A) Schematics of viral binding, entry, replication, and release assay. (**B and C**) For binding assay, Marc-145 cells were cultured at 4°C for 2 h, then infected with CHR6 strain (MOI = 5), and treated with different concentrations (0, 200, 300, 500 nM) of tubercidin. Cells were harvested after incubation at 4°C for 4 h. (B) Viral ORF7 mRNA expression was determined by RT-qPCR. (C) PRRSV particles attaching to the surface of cells were assessed using immunofluorescence analysis. The expression of N was marked by green fluorescence. Scale bar, 40 µm. (D) For entry assay, Marc-145 cells were incubated at 4°C for 2 h and infected with CHR6 strain (MOI = 5) for another 4 h at 4°C. Cells were incubated at 37°C for 4 h after tubercidin (0, 200, 300, 500 nM) treatment. Unbound viral particles were removed, and cells were collected for RT-qPCR. (E) Marc-145 cells were treated with or without tubercidin (500 nM) and infected with CHR6 strain for different time periods. Total RNA was extracted and collected for RT-qPCR to analyze the relative expression of viral ORF7. (F) Marc-145 cells were infected with CHR6 strain (MOI = 1) for 12 h at 37°C. The supernatants were discarded, and different concentrations of tubercidin (0, 200, 300, 500 nM) were added into the cells which were cultured at 37°C for another 12 h. The supernatants were collected for TCID_50_. Significant differences were denoted by (*) *P* < 0.05, (**) *P* < 0.01, (***) *P* < 0.001.

Tubercidin exerted the ability to restrain PRRSV proliferation. Therefore, we designed binding, entry, replication, and release assays to further assess the inhibitory effect on the stages of the viral life cycle (Fig. 3A). For the binding assay, the Marc-145 cells were incubated at 4°C for 2 h. Cells were untreated or treated with tubercidin at different concentrations (200, 300, 500 nM) and then infected with CHR6 PRRSV (MOI = 1). Cells were collected at 24 hpi to detect the expression of PRRSV N. As shown in Fig. 3B, there was no noticeable difference in the mRNA levels of PRRSV N. Similarly, as provided in Fig. 3C, the intensity of fluorescence of N protein presented no apparent change when compared to the cells treated without tubercidin. These data indicated that tubercidin did not influence the attachment stage of PRRSV life cycle. For the internalization assay, cells were incubated at 4°C for 2 h and infected with CHR6 strain (MOI = 5) for another 4 h at 4°C. Next, Marc-145 cells were incubated at 37°C with tubercidin (0, 200, 300, 500 nM) for 4 h allowing viral entry. Cells were harvested to examine the mRNA expression levels using RT-qPCR. As shown in Fig. 3D, tubercidin apparently decreased PRRSV N mRNA expression, suggesting its ability to block viral internalization. For the replication assay, the PRRSV N mRNA relative expression (Fig. 3E) was significantly declined at 24 and 36 hpi in the presence of tubercidin compared with the cells without drug treatment. For the release assay, different concentrations of tubercidin (0, 200, 300, 500 nM) were added into Marc-145 cells, which were infected with CHR6 strain for 12 h. The supernatants of the cells were collected for TCID_50_ after the cells were cultured for another 12 h at 37°C. The results showed that tubercidin notably reduced the viral titer, suggesting that it inhibited the viral release stage (Fig. 3F). The above data displayed that tubercidin was able to block PRRSV internalization, replication, and release of the viral life cycle.

### Tubercidin blocks PRRSV replication by promoting the activation of RIG-I/NF-κB signaling pathway

**Fig 4:**
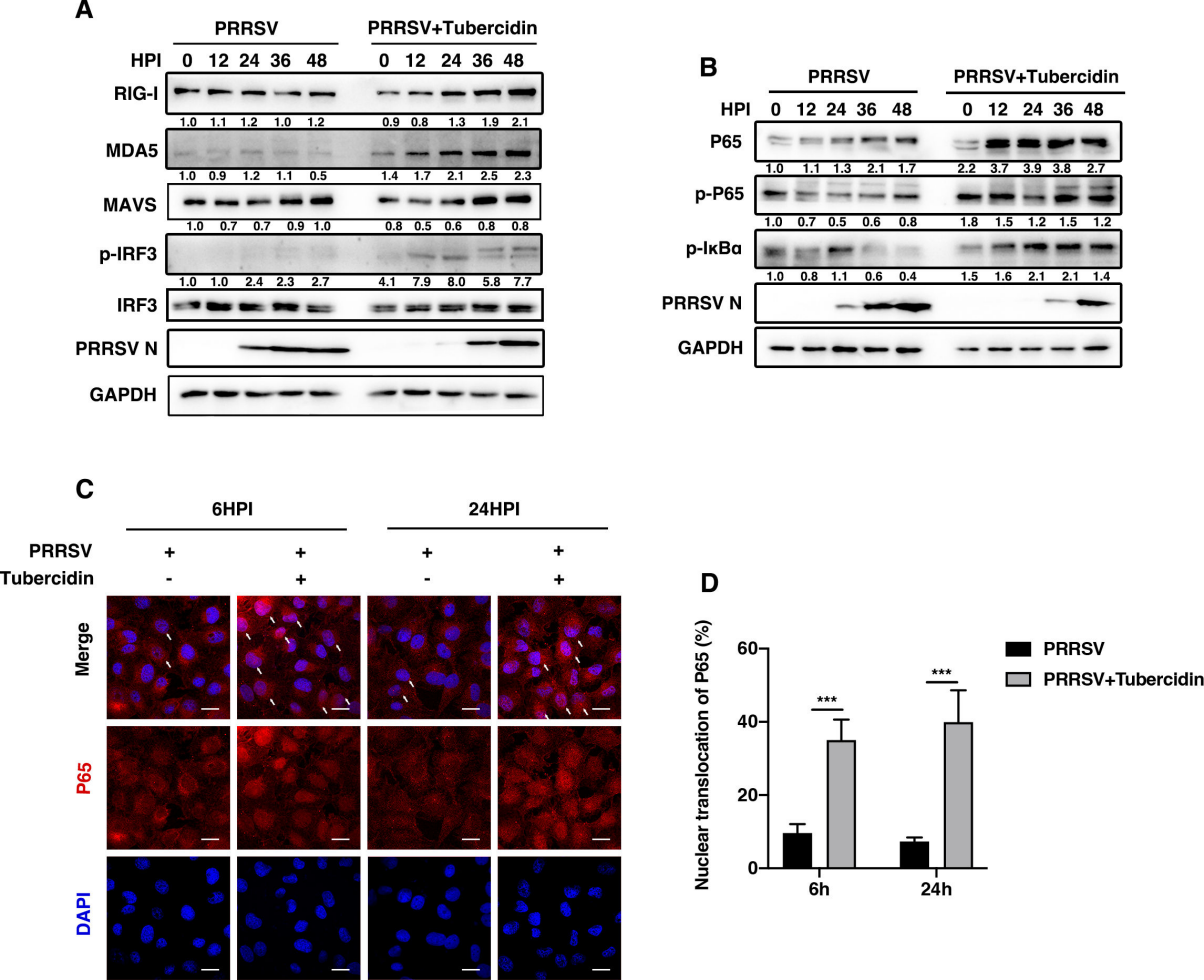
Tubercidin blocks PRRSV replication by promoting the activation of the RIG-I/NF-κB signaling pathway. (**A and B**) Marc-145 cells were mock treated or treated with tubercidin for 3 h and then infected with CHR6 strain (MOI = 1) for different time periods. RIG-I, MDA5, MAVS, phosphorylated IRF3, IRF3, p65, phosphorylated p65, phosphorylated IκBα, and PRRSV N were detected by specific antibodies using western blot at 0, 12, 24, 36, and 48 hpi. GAPDH was used as an internal control. (C) Marc-145 cells were treated in the absence or presence of tubercidin and then infected with CHR6 strain (MOI = 1) for 6 and 24 h. Cells were harvested to investigate the nuclear translocation of p65 (red) using confocal microscopy analysis. Nuclei were stained with DAPI (blue). White arrows represent the nucleus translocation of p65. Scale bar, 20 µm. (D) Quantification of NF-κB p65 translocation based on the visual images in (C). A total of ~300 cells were present for each time point. Significant differences are denoted by (***) *P* < 0.001.

The RIG-I and melanoma differentiation-associated gene 5 (MDA5) are responsible for RNA virus recognition and type I interferon (IFN) production, which is a key step in response to the virus infection (24). To investigate whether tubercidin treatment could affect RIG-I signaling, Marc-145 cells were mock treated or treated with tubercidin and then infected with PRRSV at the indicated time points. As shown in Fig. 4A, the expression of RIG-I and MDA5 was increased at 24, 36, and 48 hpi in the presence of tubercidin. Compared with the control group, the phosphorylation of IRF3 was consistently augmented at 12, 24, 36, and 48 hpi. It is known that the activation of RIG-I and MDA5 can stimulate the downstream NF-κB pathway and pro-cytokine response. We conducted western blot and confocal microscopy assays to explore whether tubercidin could activate NF-κB signaling pathway. As shown in Fig. 4B, the expression of p65 was increased at 12, 24, 36, and 48 hpi, and the level of p65 phosphorylation was enhanced at 12, 24, 36, and 48 hpi compared to the cells without tubercidin treatment. The phosphorylation of IκBα was also magnified, resulting in the translocation of p65 from the cytoplasm to the nucleus and triggering the target gene transcription. We observed that p65 (red) in the nucleus was boosted at 6 and 24 hpi when cells were treated with tubercidin (Fig. 4C and D). Taken together, tubercidin could promote the activation of RIG-I/NF-κB signaling pathway upon PRRSV replication.

### Tubercidin facilitates the expression of IFN-β, ISG56, and proinflammatory cytokines

**Fig 5:**
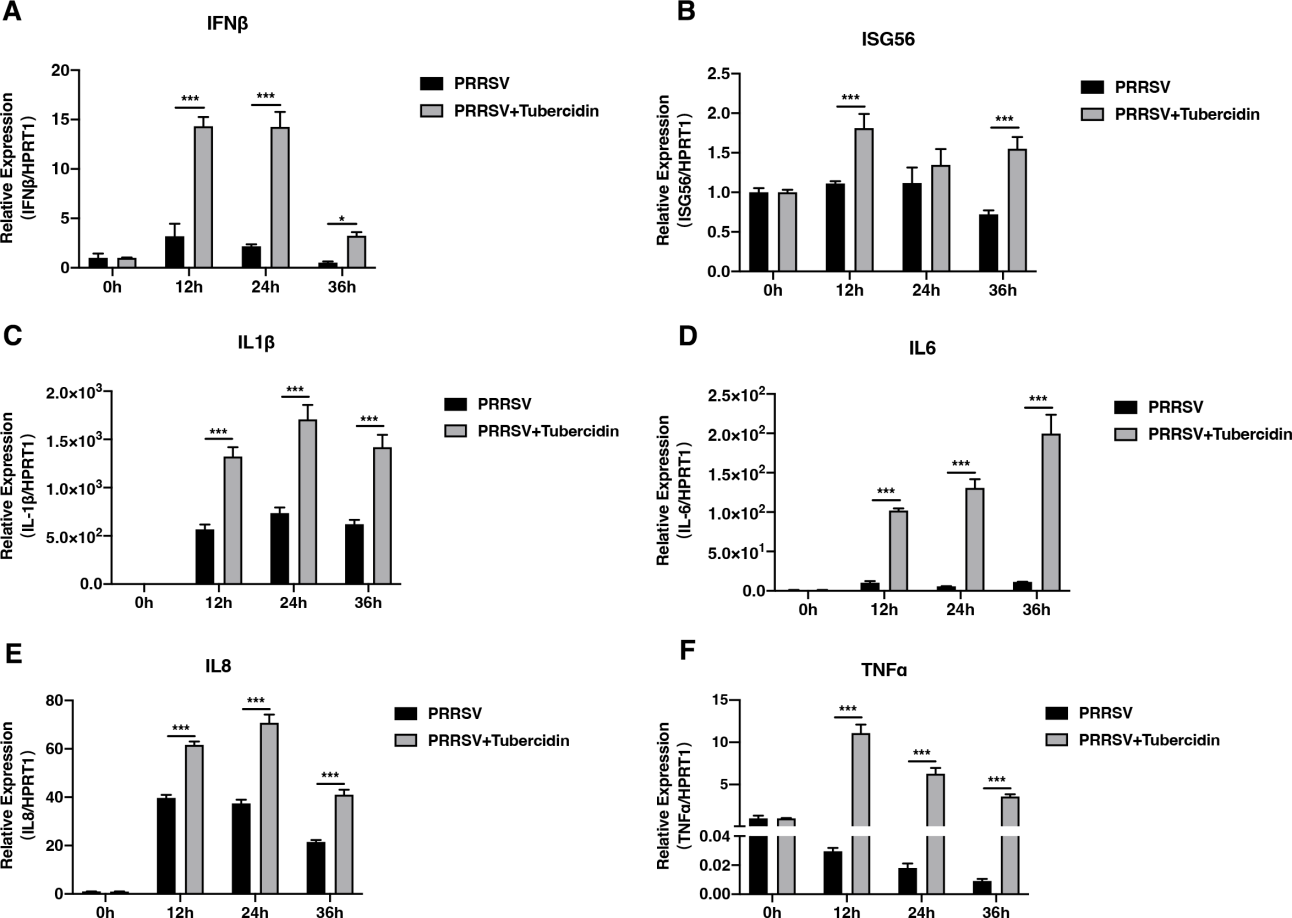
Tubercidin facilitates the expression of IFN-β, ISG56, and proinflammatory cytokines. (**A through F**) PAMs were mock infected or infected with CHR6 strain (MOI = 1) in the absence or presence of tubercidin for 0, 12, 24, and 36 h. The expression of IFN-β (A), ISG56 (B), IL-1β (C), IL-6 (D), IL-8 (E), and TNF-α (F) was analyzed by RT-qPCR. HPRT1 served as an internal control. Significant differences were denoted by (*) *P* < 0.05, (**) *P* < 0.01, (***) *P* < 0.001.

To explore whether tubercidin could affect the production of cytokines that respond to the activation of RIG-I/NF-κB signaling pathway, PAMs were infected with PRRSV in the absence or presence of tubercidin treatments (500 nM), and the relative expression of inflammatory cytokines, IFN-β, and ISG56 was assessed using RT-qPCR. Compared with the controls, the mRNA levels of IFN-β were significantly increased at 12 and 24 hpi and slightly increased at 48 hpi (Fig. 5A). Additionally, the expression of ISG56 at the transcription level was elevated at 12 and 36 hpi in the tubercidin-treated cells (Fig. 5B). Furthermore, the relative expression of IL-1β, IL-6, IL-8, and TNF-α had a significant increase at 12, 24, and 36 hpi when cells were treated with tubercidin (Fig. 5C through F). These results revealed that tubercidin promoted the expression of IFN-β, ISG56, and proinflammatory cytokines to inhibit PRRSV replication.

### Tubercidin inhibits the synthesis of PRRSV nsp2 and dsRNA to exert its antiviral ability

**Fig 6:**
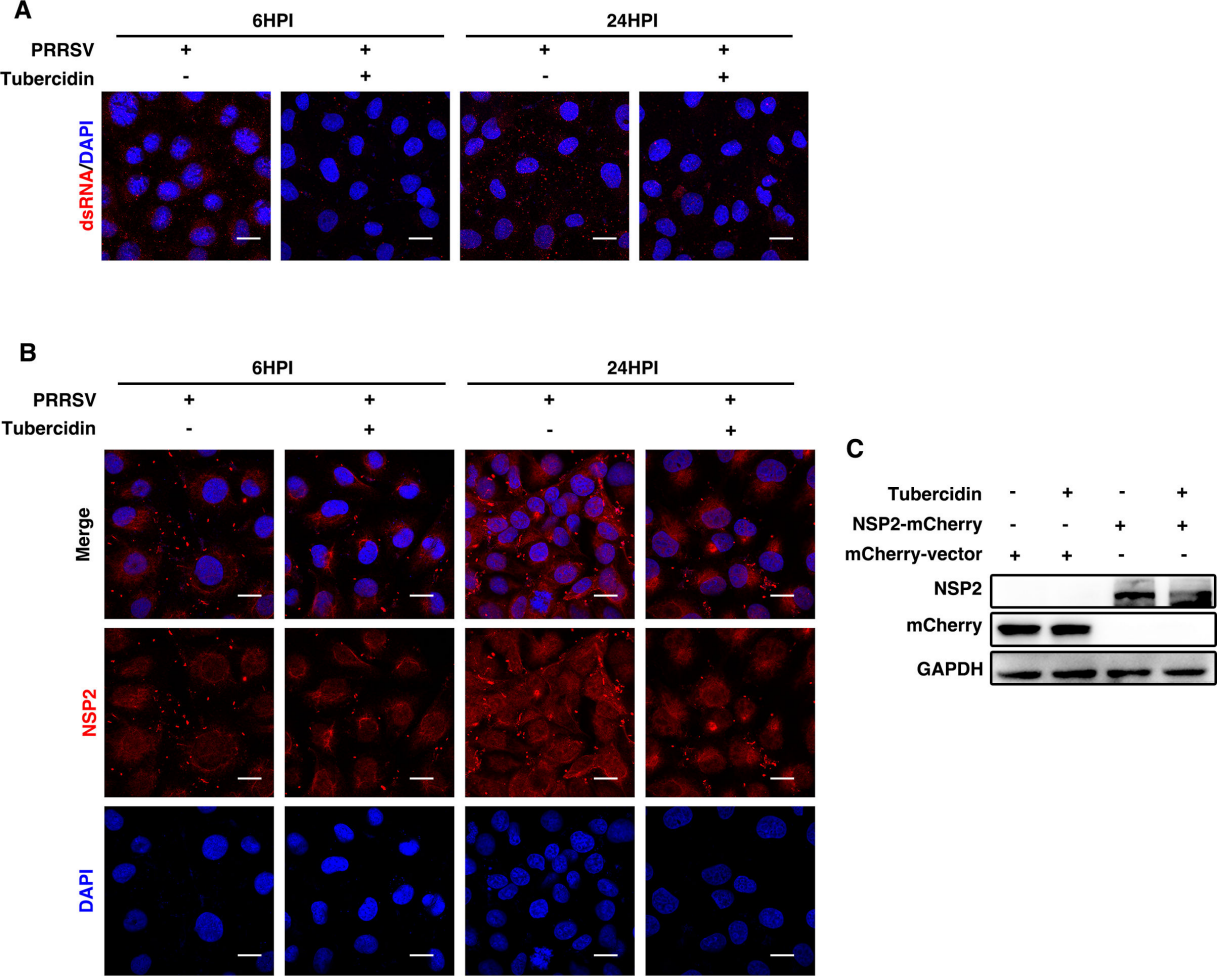
Tubercidin inhibits the synthesis of PRRSV nsp2 and dsRNA to exert its antiviral ability. (**A and B**) Marc-145 cells mock treated and treated with tubercidin were infected with CHR6 strain (MOI = 1). Immunofluorescence analysis was performed to detect the expression of dsRNA and nsp2 using specific antibodies. DAPI was used to mark the nucleus. Scale bar, 20 µm. (C) HEK293T cells were transfected with the vector plasmid or plasmid expressing mCherry-tagged nsp2 in the presence or absence of tubercidin, respectively. Cells were harvested at 24 h for western blot using anti-mCherry and anti-GAPDH antibodies.

We have identified that tubercidin displayed a strong ability to block the replication stage of the viral life cycle. To investigate the underlying mechanism, we first investigated whether dsRNA formation was blocked using the confocal microscopy assay. As presented in Fig. 6A, the fluorescence of dsRNA (red) was significantly reduced at 6 and 24 hpi in the cells treated with tubercidin, suggesting that tubercidin decreased viral RNA production. Next, we analyzed the effect of tubercidin on nsp2, an important component viral protein of RTC. Marc-145 cells were infected with PRRSV in the absence and presence of tubercidin. Cells were collected at 6 and 24 hpi for immunofluorescence analysis. As shown in Fig. 6B, the expression of nsp2 was diminished after tubercidin treatment at both 6 and 24 hpi. Meanwhile, HEK293T cells were transfected with vector plasmid or mCherry-tagged nsp2 plasmids and then mock treated or treated with tubercidin. Western blot was used to detect the expression of nsp2. As expected, we found that the expression of nsp2 protein was decreased by the tubercidin treatment (Fig. 6C). These data indicated that tubercidin exerted its antiviral ability via restricting the expression of nsp2 and then blocked viral dsRNA synthesis.

## DISCUSSION

**Fig 7:**
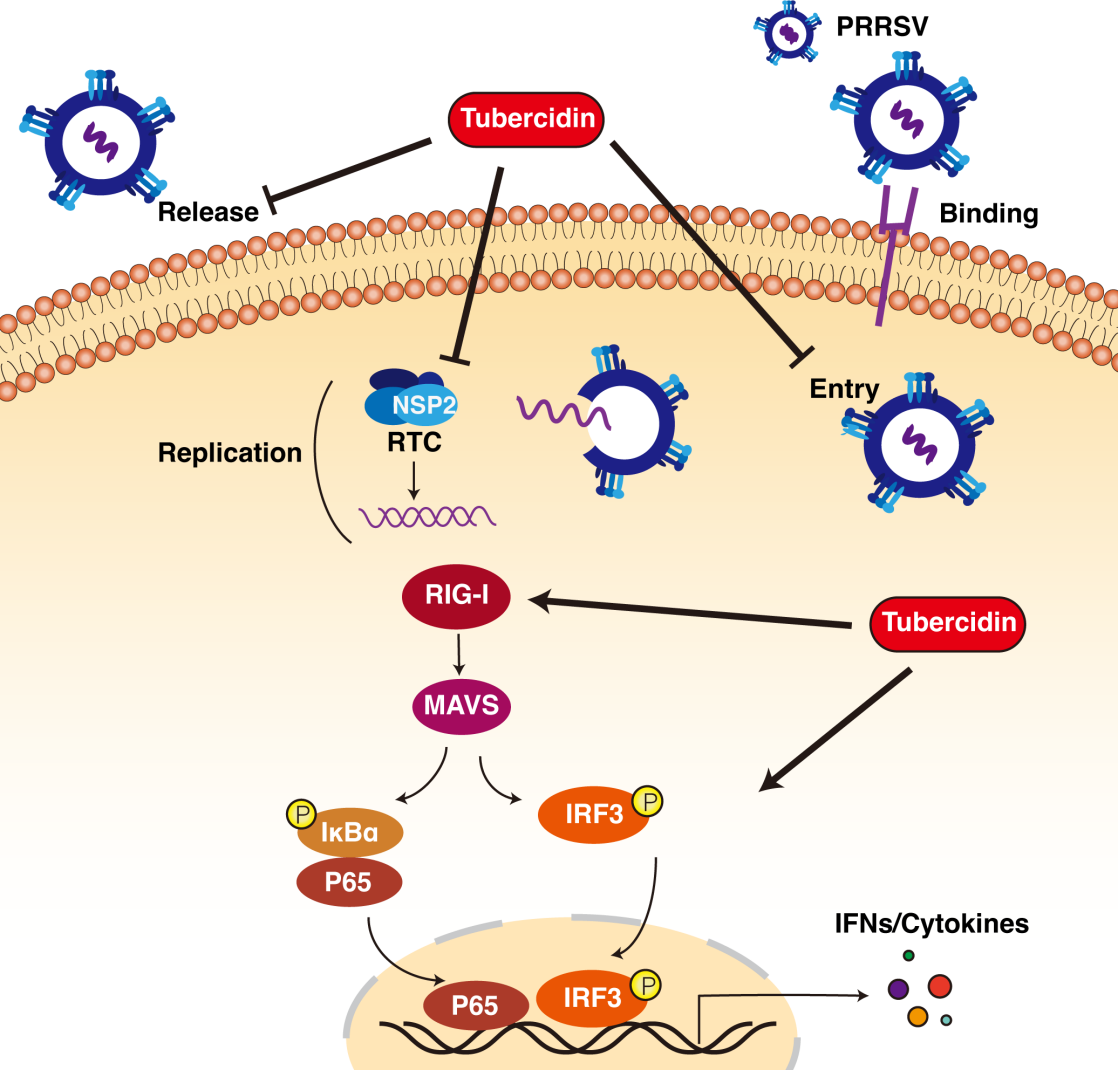
Schematic model of the inhibitory ability of tubercidin against PRRSV. Tubercidin blocked three steps of viral life cycle, including binding, replication, and release, and decreased the nsp2 expression to inhibit PRRSV replication. Additionally, tubercidin could promote the activation of RIG-I/NF-κB signaling pathway and increase the expression of IFNs and proinflammatory cytokines, leading to PRRSV inhibition.

PRRSV severely impacts the swine industries and causes huge economic losses for years. Because of the inadequate understanding of PRRSV virological and recombination mechanisms, development of effective vaccines is still on the way (25). In recent years, increasing research interests have focused on the promising antiviral roles including host microRNAs, herbal extracts, chemical substances, and nanobodies (26). Here, our study shows that tubercidin, a natural origin compound, has the potential to inhibit PRRSV replication via multiple approaches as shown in Fig. 7. We determined that tubercidin interrupted the internalization, replication, and release steps of the PRRSV life cycle. Moreover, we illustrated that tubercidin inhibited nsp2 expression and enhanced the activation of RIG-I/NF-κB signaling to exert the antiviral ability against PRRSV replication.

PRRSV infection induces the formation of double-membrane vesicles (DMVs), derived from the cellular endoplasmic reticulum, which provide an ideal microenvironment for viral RNA replication (27). Meanwhile, the aggregate viral replicase enzymes and partial cellular proteins are assembled as a replication and transcription complex (RTC), which is associated with the DMVs and produces viral RNA genome and sg mRNAs. PRRSV nsp2 plays an essential role in RTC, viral RNA formation, and viral replication. Our results identified that tubercidin suppressed viral dsRNA synthesis (Fig. 6A). We then further explored the underlying mechanism and interestingly found that tubercidin downregulated nsp2 expression. There were three possible reasons to explain that decreased nsp2 inhibited viral dsRNA synthesis. First, nsp2 is responsible for cleaving the nsp2–3 junction and activating nsp4 to process the remaining nsps, which are indispensable for RTC (28). Second, nsp2 induces the rearrangement of the RTC-related membranes and modifies the formation of the DMVs (29). Besides, it was revealed that nsp2 interacted with several nsps, containing nsp1α, nsp1 β, nsp3, nsp4, nsp7, nsp9, and nsp10, which were crucial replicase proteins in viral RTC (30). Tubercidin was reported to be incorporated into viral RNA, which suppresses several biochemical pathways including protein synthesis (31, 32). Certainly, we speculated that tubercidin had the possibility to inhibit the synthesis of other nsps. Nevertheless, the specific effects of tubercidin on viral proteins need to be investigated in future studies. In addition, it is doubtful whether tubercidin directly affects the synthesis of nsp2 or degrades the synthesized nsp2. Due to its function of inhibiting protein synthesis, we are prone to think that tubercidin inhibits the nsp2 synthesis, thus decreasing its expression. Collectively, tubercidin suppressed nsp2 expression and dsRNA synthesis, ultimately inhibiting PRRSV replication.

Besides inhibiting viral replication, our results showed that tubercidin also blocked the entry and release stages of viral life cycle. PRRSV has a restricted cell tropism due to the interaction between cellular receptors and viral envelope proteins. Previous studies have revealed that GP2 and GP4 interact with the scavenger receptor CD163. which is indispensable for PRRSV entry and release (28, 33). Moreover, it has been reported that GP5, along with M, is required for viral release (34). Therefore, we speculate that tubercidin could block PRRSV internalization and release by suppressing the synthesis of related proteins, including GP2, GP4, GP5, and M, or interrupt the interaction between viral proteins and host receptors.

Innate immunity is the first barrier for the host to defend against pathogenic microorganisms by recognizing them and transducing a signaling cascade to activate downstream effector molecules to exert antiviral immune functions. The RIG-I-like receptor family, including RIG-I and MDA5, is one of the five pattern-recognition receptor families (35). RIG-I and MDA5 are essential RNA sensors with their carboxy-terminal domain sensing short and long dsRNA, respectively (36). RIG-I and MDA5 interact with the adaptor MAVS to promote the phosphorylation of the interferon regulatory factor 3 (IRF3) by TANK-binding kinase 1 and IκB kinase-ε. IRF3 and NF-κB are essential transcription factors and translocate into the nucleus to induce the production of IFN-β and proinflammatory cytokines such as IL-1, IL-6, IL-8, and TNF-α to defend against PRRSV infection. Besides, the phosphorylation of IκBα, which remains the dimers of p50 and p65, is required for the activation and translocation of NF-κB (37). The type I IFN signaling pathway plays a critical role in defending the viral infection and induces the transcription of IFN-stimulated genes (ISGs), which have direct antiviral effects (38). Our findings demonstrated that tubercidin could activate the RIG-I and MDA5 expression in Marc-145 cells. Additionally, tubercidin treatment stimulated the phosphorylation of IRF3, IκBα, and p65 to promote the translocation of IRF3 and p65 and transcription of IFN-β, ISG56, and inflammatory cytokines to repress PRRSV replication *in vitro* (Fig. 4 and 5). Considering the immune-stimulating effects of tubercidin, it possesses potential value as an adjuvant, which should be further examined both *in vitro and in vivo*. Interestingly, tubercidin is one of the naturally existing 7-deazapurines, which were reported to be a privileged scaffold for designing novel antiviral nucleosides. Many 7-deazapurine nucleosides have been artificially synthesized with alterations of the nucleoside sugar moiety and substituents of C7 and C8 (39). In addition, multiple promising antitumor and antiviral compounds have been synthesized, such as 7-hetaryltubercidin and trifluoromethyl-tubercidin (15, 40). Therefore, it is possible to generate modifiable tubercidin-related compounds with more effective antiviral capability and less cytotoxicity. Importantly, clinical trials need to be carried out to evaluate the efficiency of tubercidin *in vivo,* and there are still many obstacles to clinical application, such as the dose of administration, the animals, the poor economic benefit, and many unknown factors.

In conclusion, our study showed that tubercidin could inhibit three steps of the viral life cycle: internalization, replication, and release. Furthermore, it blocked the nsp2 expression, which is a crucial replicase protein in viral replication, and stimulated the RIG-I/NF-κB signaling pathway to increase the antiviral cytokine production, leading to the suppression of PRRSV replication *in vitro*. Our work provides a novel experimental way for blocking PRRSV replication.
